# Arthroscopic fixation of humeral greater tuberosity fracture using a W-shaped suture

**DOI:** 10.1186/s13018-020-02077-8

**Published:** 2020-11-23

**Authors:** Xiaocong Lin, Xiuxi Huang, Kaibin Fang, Zhangsheng Dai

**Affiliations:** Department of Orthopaedic Surgery, The Second Affiliated Hospital of Fujian Medical University, No.34, Zhongshanbeilu, Quanzhou, 36200 Fujian China

**Keywords:** Fracture of greater tuberosity of humerus, W-shaped suture, Arthroscopy

## Abstract

**Background:**

Patients with greater tuberosity fractures of the humerus often require surgery. Therefore, there is a need to find a minimally invasive and effective surgical procedure with great patient outcomes.

**Aim:**

To evaluate the clinical outcomes of the W-shaped suture technique under shoulder arthroscopy in the treatment of greater tuberosity fractures of the humerus.

**Methods:**

In this retrospective study, a total of 17 patients were included. The fractures were closed, and there was no neurovascular injury. These patients underwent arthroscopically assisted reduction and internal fixation of the greater tuberosity fractures. Fixation was performed using sighting nails combined with a W-shaped suture. The imaging data of the patients were collected, and the ASES score, Constant-Murley score, and VAS score were used to evaluate the patient’s outcome. At the last follow-up (at least 1 year), the range of motion in the affected shoulder was compared with that of the contralateral side.

**Results:**

The operation was successful in all the patients. The average follow-up time was 13 months. There were no reported complications such as fracture displacement, nonunion, and internal fixation failure during the follow-up period. Post-operative X-ray examinations revealed good function recovery, with a healing time of between 10 and 12 weeks, and an average healing time of 11.5 weeks. Following the operation, patients reported reduced shoulder joint pain that no longer influenced their activity or caused discomfort in their daily life. The patient’s VAS score ranged from 0 to 3, with an average of 0.52 ± 0.73, while at the last follow-up, the Constant-Murley score ranged from 83 to 97, with an average of 92.33 ± 7.55. The ASES score ranged from 81 to 98, with an average of 93.15 ± 6.93. At the last follow-up, there was no significant difference in the overall range of motion with the unaffected limb.

**Conclusion:**

This study demonstrates that the W-shaped suture can be used to effectively fix the fractures of the greater tuberosity of the humerus, by increasing the fixed area to promote healing.

## Background

The greater tuberosity fracture of the humerus is one of the common proximal humerus fractures among the young and male population [1]. The arm of the rotator cuff tendon is destroyed following a fracture of the greater tuberosity of the humerus. The displacement of the fracture block reduces the subacromial space, which can cause the shoulder to hit as it abducts, hence affecting the shoulder joint function [2]. Therefore, when the fracture appears displaced, reduction and internal fixation of the greater tuberosity fractures are necessary. Open reduction and the cannulated screw fixation technique have been widely used to treat a displaced greater tuberosity fracture [3, 4]. However, cannulated screw fixation of the greater tuberosity fracture is associated with increased morbidity due to comminution or migration of the fractured fragment and poor fixation.

With the recent development of arthroscopy, arthroscopic treatment of humeral greater tuberosity fracture has been widely used [5]. The arthroscopy offers better visualization and mobilization of the fragment as well as treatment of any associated intra-articular pathology. Many materials have been used to fix the fracture, including cannulated screws, Kirschner wires, plates, and so on [2, 6, 7]. However, if the fracture block is small or the fracture is seriously crushed, these materials cannot achieve accurate reduction and strong fracture effect. The appearance of the wire anchor provides new ideas into the treatment of fractures [8]. In this study, we retrospectively evaluated the clinical outcome of the fixation of humeral greater tuberosity fracture using the W-shaped suture technology.

## Methods

**Fig. 1:**
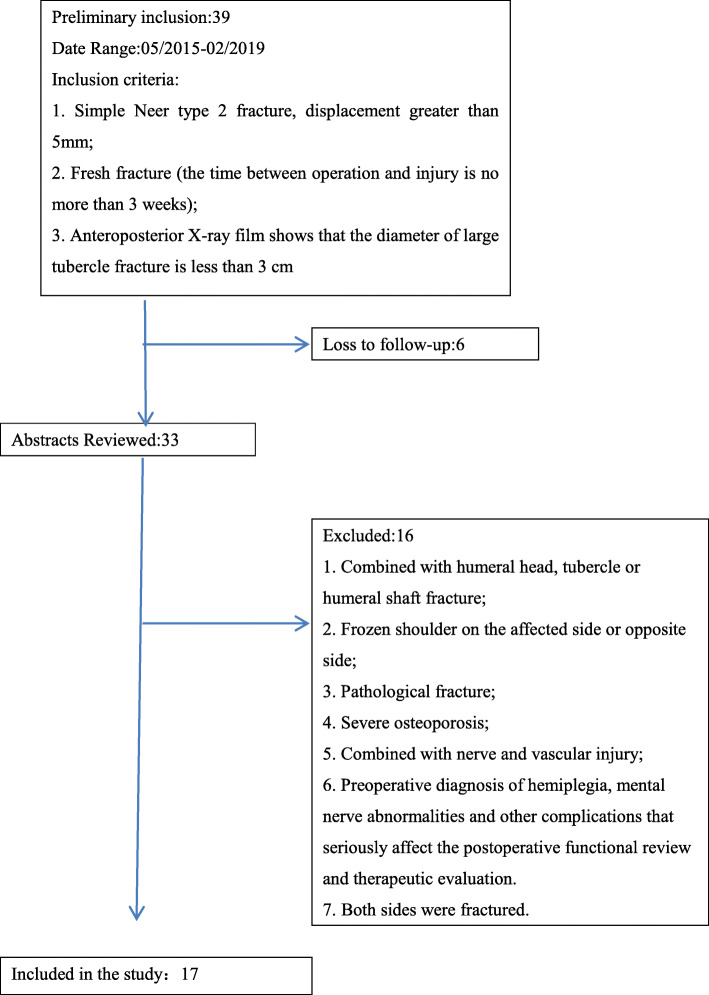
Inclusion and exclusion criteria

A total of 17 patients with humeral greater tuberosity fracture treated with arthroscopic-assisted reduction and W-shaped suture fixation at our hospital between May 2015 and February 2019 were included in this retrospective study. The inclusion and exclusion criteria are as shown in Fig. 1. Among the patients, 11 were men and 6 were women, and their average age was 53.36 years old. The time from injury to operation was 2–9 days (average 5 days). The patients showed limited movement of the affected shoulder joint with pain before the operation; however, blood supply and sensation of the affected limb were normal. Before the operation, the VAS score of the patients ranged from 8 to 4, with an average of 6.74 ± 2.16; the Constant-Murley score ranged from 74 to 58, with an average of 67.62 ± 8.35, and the ASES score ranged from 69 to 48, with an average of 58.63 ± 9.48.

**Fig. 2:**
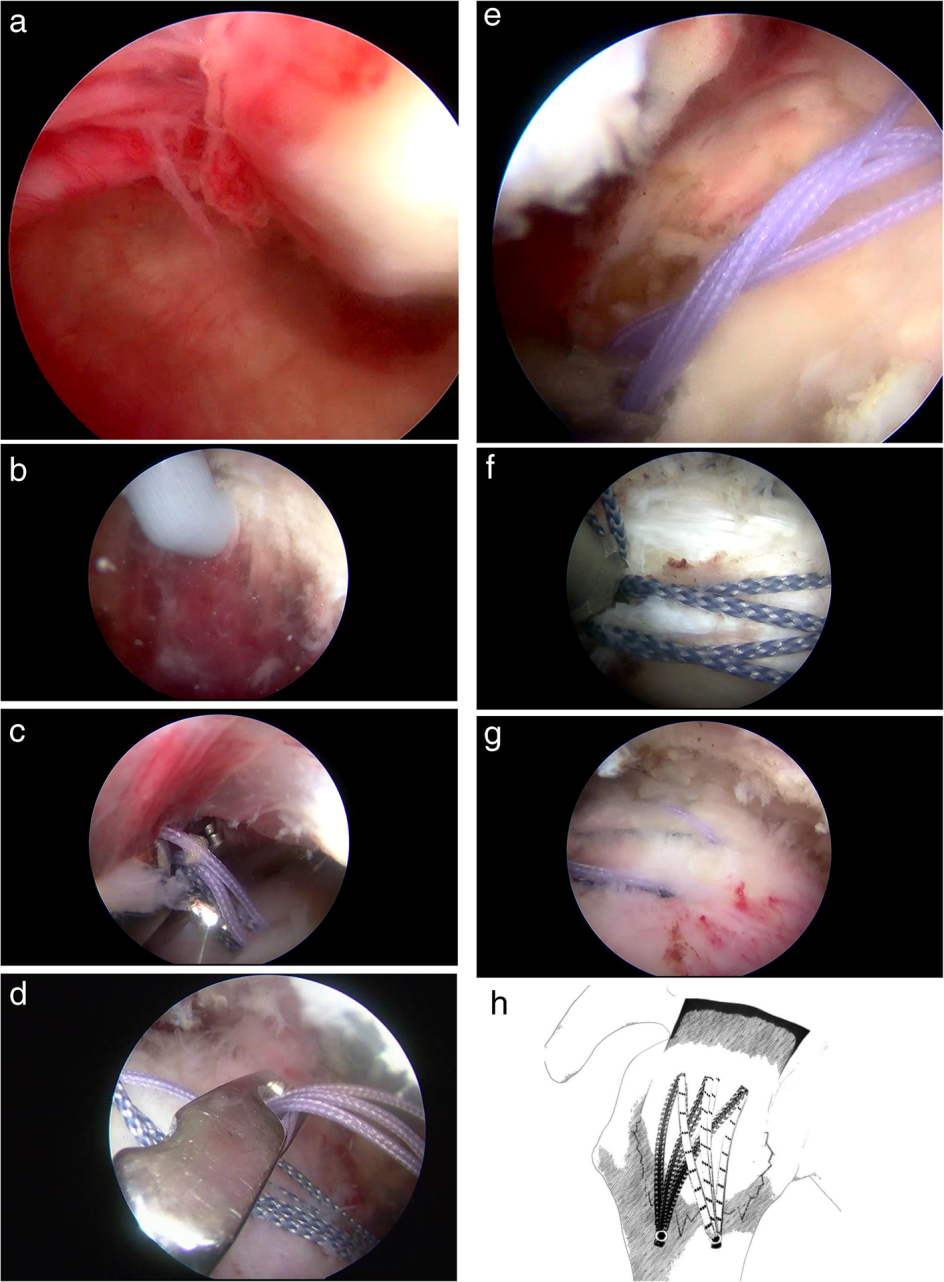
Surgical procedure (the pictures are attached). **a** Obvious displacement of the fracture seen during exploration. **b** Cleaning of the humeral surface. **c** Two stitches through the sleeves. **d** Grabbing of the same color of stitches. **e** After reduction, 4 sutures are fixed with an external row of nails. **f** The second outer row nail is used to fix the other four sutures. **g** Complete fixation. **h** Diagram

**Fig. 3:**
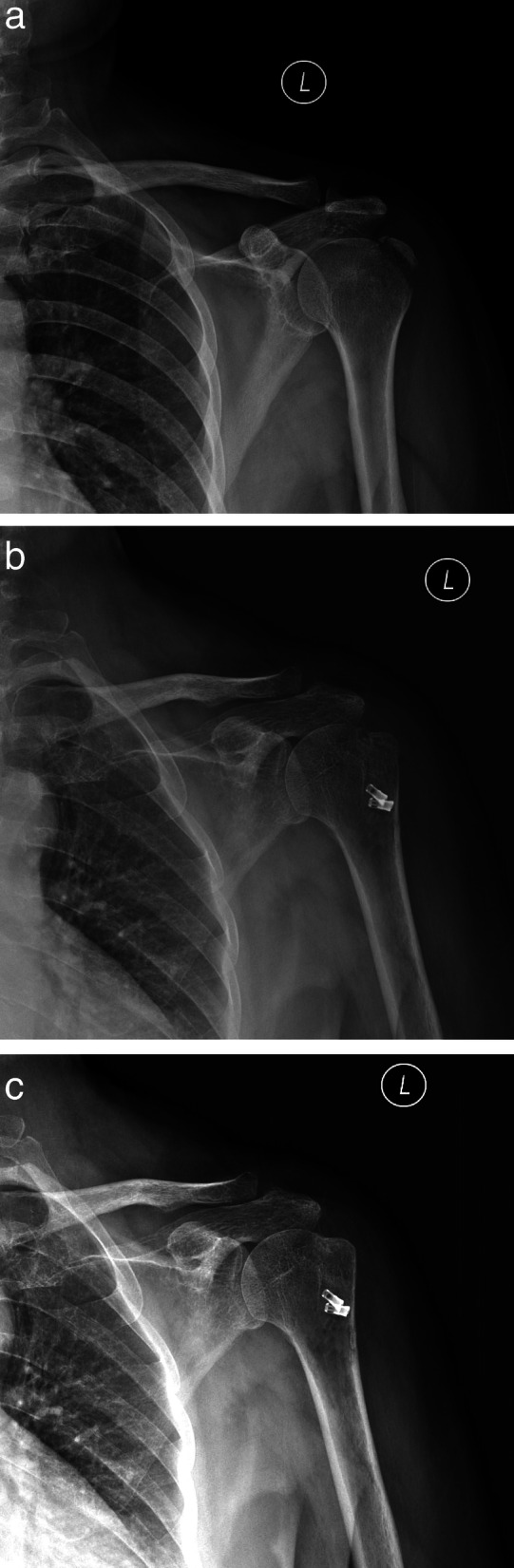
**a** Pre-operative X-ray. **b** Postoperative X-ray examination. This shows that the fracture reduction was good. **c** Fracture healing

With the patient in the lateral decubitus position, routine examination of the intra-articular joint was performed from both the posterior and anterior portals, and the displaced greater tuberosity fragment attached to the supraspinatus detected. Debridement of the edge of the fragment and crater of the fracture was performed with a shaver. The arthroscope was moved into the subacromial space, and a bursectomy was performed, particularly around the lateral margin of the fracture and the adjacent intact metaphyseal cortex, to improve visualization and ease the insertion of the anchor. The supraspinatus tendon was confirmed to have no injury and in good continuity under arthroscopy. The scope was moved to the posterolateral portal to obtain a better view of the fragment. Three points were identified at the supraspinatus tendon–tuberosity junction, including the anterior point (A), the midpoint (B), and the posterior point (C). All three points covered the entire fracture fragment. A BirdBeak suture passer (ConMed Linvatec, Largo, FL, USA) punched through the full thickness of the supraspinatus tendon–tuberosity junction at 1–2 mm posterior to the most anterior part of the fragment from point A to point B, and passed a polydioxanone suture, which was used as the suture shuttle for two Orthocord-braided sutures. Subsequently, two Orthocord sutures were passed and retrieved through the anterior cannula. The second set of two Orthocord sutures were inserted from point B to point C through the posterior portal using a similar technique. In total, 4 Orthocord sutures passed through the supraspinatus. The Orthocord suture from point A to point B and from point B to point C was retrieved as a group through the lateral cannula, and another set of Orthocord suture from point A to point B and from point B to point C were retrieved (see Fig. 2). The fracture fragment was reduced with an arthroscopic hook, and the two groups of sutures anchored into proximal humeral from anterior to posterior using two Versalok anchors (DePuy Mitek, Raynham, MA, USA) for the suture-bridge technique. The insertion points of the lateral anchors were more than 5 mm from the fracture margin to prevent cracking of the cortex and loosening the anchors. The degree of compression was adjusted under direct visualization. A second Versalok anchor was inserted at 1.0 cm posterior as previously described. The reduction of the fragment could be seen from the articular and bursal surfaces and was confirmed using post-operative radiographs (see Fig. 3).

**Fig. 4:**
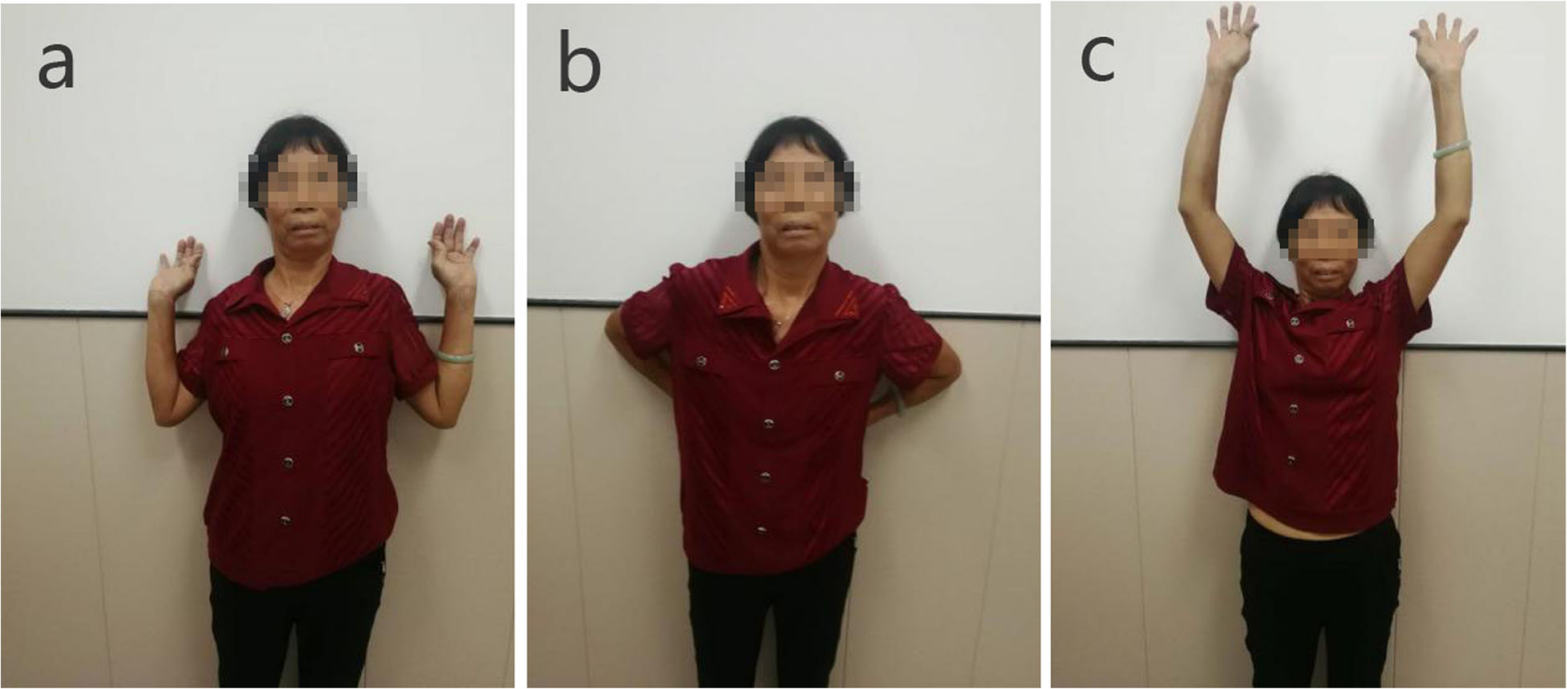
Shoulder movement ranges. **a** External rotation. **b** posterior extension. **c** Elevation

The patients received wound cleaning and an X-ray examination on the first day following the operation. Besides, the follow-ups were at 4 weeks, 12 weeks, 6 months, and 12 months. After the operation, the affected limb was kept at 30° of abduction using a shoulder brace to avoid the displacement of the fracture end, which can be caused by traction of the supraspinatus muscle. After 3 weeks, the patients were allowed to do passive exercise. Based on the X-ray examination results of the shoulder joint, patients were allowed to participate in active exercise, about 6 weeks after the operation. Following the identification of fracture healing by X-ray, the patients were allowed to resume full activities and weight-bearing (see Fig. 4).

The visual analog scale was used to evaluate the pain level of the patients [9]. The lower the score, the lighter the pain. American Shoulder & Elbow Surgeons score and the Constant-Murley score were used to evaluate the shoulder function. The higher the score, the better the function. At the last follow-up (at least 1 year), the range of motion of the affected shoulder was compared with that of the healthy shoulder.

### Statistical analysis

Data obtained from both the preoperative and postoperative visual analog scale (VAS) were compared using a 2-sample *t* test. A significance level of *p* < 0.05 was used, and all results were presented as mean ± standard deviation (SD).

## Results

The operation was successful in all the patients, and the operation time ranged from 45 to 95 min, with an average of 70.5 min. There were 2 patients with a subscapular muscle injury, which was repaired during the operation. None of the patients developed complications, such as wound infection, vascular damage, or nerve damage. The end of the follow-ups was determined as fracture healing or revision surgery. However, none of the patients required revision surgery, and the average follow-up time was 13 months. Postoperative X-ray film showed that the fracture was well reduced, and there was no internal fixation failure and none of the anchors had pulled out. X-ray examination of the patients revealed that all the fractures healed smoothly, and the healing time was 10–12 weeks, with an average of 11.5 weeks. Following the operation, patients reported reduced shoulder joint pain that no longer affected their activity or caused discomfort in their daily life. The patient’s VAS score ranged from 0 to 3, with an average of 0.52 ± 0.73, while at the last follow-up, the Constant-Murley score ranged from 83 to 97, with an average of 92.33 ± 7.55. The ASES score ranged from 81 to 98, with an average of 93.15 ± 6.93. The VAS score, the Constant-Murley score, and the ASES score were significantly improved after surgery (see in Table 1). At the last follow-up, there was no significant difference in the overall range of motion with the unaffected limb (see in Table 2).

**Table 1:** The VAS score, the Constant-Murley score, and the ASES score

	Preoperative (*n* = 20)	Postoperative (*n* = 20)	Statistic (*T* value/χ^2^ value)	*p* value
VAS score	6.74 ± 2.16	0.52 ± 0.73	12.20	0.00
Constant-Murley score	67.62 ± 8.35	92.33 ± 7.55	9.82	0.00
ASES score	58.63 ± 9.48	93.15 ± 6.93	13.15	0.00

**Table 2:** Comparison of shoulder range of motion between the affected side and the healthy side

Variable	Affected side (*n* = 20)	Healthy side (*n* = 20)	Statistic (*T* value/χ^2^ value)	*p* value
Abduction	81.36 ± 6.77	83.26 ± 7.35	0.85	0.40
External rotation	53.26 ± 5.79	55.12 ± 6.33	0.97	0.34
Internal rotation	59.13 ± 4.37	60.35 ± 5.55	0.77	0.44
Adduction	31.23 ± 6.59	33.15 ± 7.13	0.88	0.38
Anteflexion	80.12 ± 6.55	82.36 ± 7.63	0.99	0.33
Extension	35.06 ± 3.92	36.15 ± 5.92	0.69	0.49

## Discussion

The indication of the fracture of greater tuberosity of the humerus fractures is controversial. The greater tuberosity of the humerus is the insertion point of the supraspinatus muscle. Poor reduction after fracture significantly increases the abduction strength of the shoulder joint provided by the deltoid muscle [9]. Some scholars believe that surgical treatment should be considered when the fracture displacement is greater than 5 mm, and conservative treatment should be considered when the fracture displacement is less than 5 mm [10]. For young patients with fracture displacement, less than 5 mm and greater than 3 mm, athletes, and other patients with high requirements for shoulder joint function, surgical treatment is recommended [11]. Anatomical reduction of the greater tuberosity fracture is highly important. If the fracture block is fixed to a lower position, the traction force of the rotator cuff to the large tubercle increases, leading to failure of fixation and the limitation of shoulder joint function, which may lead to secondary rotator cuff injury, and acromion impingement syndrome may also occur postoperatively [10].

Arthroscopy allows the surgeon to directly observe the location of the fracture site and can also cause potential rotator cuff injury [12]. Compared with open surgery, arthroscopy reduces scar formation and the incidence of shoulder joint adhesion and minimizes the impact on shoulder joint function [13]. Many scholars use the arthroscopic suture bridge technique to treat humeral greater tuberosity fracture [5]. Compared with cannulated screw fracture, the suture bridge technique can effectively utilize the traction force of supraspinatus muscle to the greater tuberosity bone mass, to stabilize the fracture. We developed the W-shaped suture based on suture bridge technology, which only requires two lateral anchors, while the suture bridge technology generally requires four anchors. W-shaped suture technology saves on the cost, and the fracture block covers the footprints 100%, which not only reduces the tension of the supraspinatus muscle but also provides maximum contact area, which increases the fixation strength of the fracture block and reduces the formation of space. At the same time, the W-shaped suture technology was used to disperse the high-strength thread at the end of the anchor bolt on the fracture block, to achieve a satisfactory suture and fixation effect. The reticular structure of the high-strength line can firmly fix the avulsion bone block and rotator cuff tissue, to prevent fracture displacement. All the patients in this study achieved satisfactory fracture reduction. During the long-term follow-up, the patient’s shoulder function and range of motion were maximally restored.

For doctors who have previously experienced shoulder arthroscopy, the proposed technique will be very easy to use. However, there are several points to consider during the operation. This technique is not suitable for patients with a large diameter (> 3 cm) of nodule fracture. This is because the operation space becomes smaller and more difficult when the large tubercle fracture block is large, and this may increase the risk of axillary nerve injury [14, 15]. The average distance from the sub-acromial to the axillary nerve is about 6 cm [16]. Therefore, the transverse mark should be made 5 cm below the acromion before an operation [17], and the lateral mark should not be exceeded during the operation to avoid injuring the axillary nerve.

### Limitations

The present study has a few limitations. First, the sample size in this study is small. Second, the study does not compare the proposed technology with other technologies in the field. Therefore, future studies should address the present limitations to validate the findings and obtain more comprehensive results.

## Conclusion

This study demonstrates that the W-shaped suture technique can be used to effectively fix the fracture of the greater tuberosity of the humerus. This technique increases the fixed area to promote healing; hence, it is an effective treatment method.

## Data Availability

The datasets used and/or analyzed during the present study are available from the corresponding author upon reasonable request.
